# Neurodegeneration, Myelin Loss and Glial Response in the Three-Vessel Global Ischemia Model in Rat

**DOI:** 10.3390/ijms21176246

**Published:** 2020-08-28

**Authors:** Tatiana Anan’ina, Alena Kisel, Marina Kudabaeva, Galina Chernysheva, Vera Smolyakova, Konstantin Usov, Elena Krutenkova, Mark Plotnikov, Marina Khodanovich

**Affiliations:** 1 Laboratory of Neurobiology, Research Institute of Biology and Biophysics, Tomsk State University, Lenina Ave., 634050 Tomsk, Russia; tany_a@list.ru (T.A.); kisell.alena@gmail.com (A.K.); kmsra08@gmail.com (M.K.); usov.konstantin1984@yandex.ru (K.U.); lenk@yandex.ru (E.K.); 2 Laboratory of Pharmacology of Blood Circulation, E. D. Goldberg Research Institute of Pharmacology and Regenerative Medicine, Tomsk National Research Medical Center, Russian Academy of Sciences, Lenina Ave., 634028 Tomsk, Russia; bona2711@mail.ru (G.C.); light061265@mail.ru (V.S.); mbp2001@mail.ru (M.P.)

**Keywords:** global cerebral ischemia, GCI, hippocampus, CA1, neurodegeneration, myelin, oligodendrocyte, inflammation, rod-shaped microglia, oligodendrogenesis

## Abstract

(1) Background: Although myelin disruption is an integral part of ischemic brain injury, it is rarely the subject of research, particularly in animal models. This study assessed for the first time, myelin and oligodendrocyte loss in a three-vessel model of global cerebral ischemia (GCI), which causes hippocampal damage. In addition, we investigated the relationships between demyelination and changes in microglia and astrocytes, as well as oligodendrogenesis in the hippocampus; (2) Methods: Adult male Wistar rats (*n* = 15) underwent complete interruption of cerebral blood flow for 7 min by ligation of the major arteries supplying the brain or sham-operation. At 10 and 30 days after the surgery, brain slices were stained for neurodegeneration with Fluoro-Jade C and immunohistochemically to assess myelin content (MBP+ percentage of total area), oligodendrocyte (CNP+ cells) and neuronal (NeuN+ cells) loss, neuroinflammation (Iba1+ cells), astrogliosis (GFAP+ cells) and oligodendrogenesis (NG2+ cells); (3) Results: 10 days after GCI significant myelin and oligodendrocyte loss was found only in the *stratum oriens* and *stratum pyramidale*. By the 30th day, demyelination in these hippocampal layers intensified and affected the *substratum radiatum*. In addition to myelin damage, activation and an increase in the number of microglia and astrocytes in the corresponding layers, a loss of the CA1 pyramidal neurons, and neurodegeneration in the neocortex and thalamus was observed. At a 10-day time point, we observed rod-shaped microglia in the *substratum radiatum*. Parallel with ongoing myelin loss on the 30th day after ischemia, we found significant oligodendrogenesis in demyelinated hippocampal layers; (4) Conclusions: Our study showed that GCI-simulating cardiac arrest in humans—causes not only the loss of pyramidal neurons in the CA1 field, but also the myelin loss of adjacent layers of the hippocampus.

## 1. Introduction

White-matter injury—along with neuronal loss—is an integral part of the pathologic processes that accompany cerebrovascular diseases. Poor white-matter recovery significantly affects the long-term outcome after an acute stroke [1], impairs sensorimotor function and causes profound neurobehavioral and cognitive impairment [2]. The main function of myelin, which is to increase the speed with which electrical impulses propagate along the myelinated fiber, is well known. Other functions of myelinating cells, including structural, metabolic and energetic support of the axon, are far less studied or have been established recently [3].

Despite its importance for normal brain functioning and recovery after stroke, demyelination has rarely been investigated in the animal models of brain ischemia. The studies on the animal middle cerebral artery occlusion (MCAO) model [4,5,6,7,8] studies showed that myelin destruction begins from the first day and even as early as hours after stroke. The mechanisms of demyelination [5,6,8] include intramyelinic edema that manifests as myelin pallor, further vacuole formation and sheath destruction followed by myelin debris clearance by microglia and macrophages. The main part of the destroyed myelin is removed by the 7–10th day after the stroke [4]. In addition, oligodendrocytes, which are extremely sensitive to ischemia, die, which complicates the remyelination of remaining axons [5,6,9]. Molecular mechanisms underlying oligodendrocyte death include glutamate excitotoxicity, mitochondrial dysfunction, oxidative stress and proinflammatory factors [10].

The brain is inherently more vulnerable to ischemia than any other organ and suffers significant damage after only a few min of complete cerebral blood flow interruption. The localization, severity of lesions, and reparative response are significantly dependent on the animal model of cerebral ischemia. The most widely used and standardized models of focal ischemia, such as the (MCAO) model, are characterized by damage to the striatum and cortex [4,11,12]. In contrast with this type of models, a common feature of the global cerebral ischemia (GCI) model is primary damage to the hippocampus [13,14,15,16,17,18,19]. These models are relatively rare, less standardized and used in several variants. There are fundamentally different GCI models of hypoxia, in which only inhibition of systemic circulation occurs and models of complete interruption of cerebral blood flow that simulates cardiac arrest in humans. Among the GCI models, only a few provide complete interruption of blood flow followed by reperfusion. A simple closure of the common carotid arteries completely interrupts the blood flow to the brain only in gerbils, which, unlike mice or rats, have an incomplete a Circle of Willis [20]. The model of GCI with complete interruption of the brain blood flow in a mouse or rat requires more comprehensive techniques.

The three-vessel model of GCI [16,17,19] used in this study provides complete interruption of the cerebral blood flow, followed by reperfusion that mimics cardiac arrest in humans and emulates possible injuries of the hippocampus after it. Changes in the hippocampus observed in rats after GCI are similar to lesions in the hippocampus in humans undergoing anoxia [21]. Thus, MRI studies demonstrate a decrease in the volume of the hippocampus in both hemispheres in patients after anoxia during cardiac arrest or an ischemic episode [22,23]. These results are confirmed by post-mortem histological studies of brain sections of patients after anoxia, which reveal a limited bilateral lesion of the hippocampus, resulting in a complete loss of pyramidal neurons of the CA1 field [24], extensive loss of pyramidal neurons of the CA3 field and partial damage to the CA2 field [25].

In humans, damage to the hippocampus caused by anoxia can lead to severe anterograde amnesia, which confirms the role of the hippocampus as a critical structure for memory function [21,23]. The pyramidal neurons of the CA1 field of hippocampus are extremely vulnerable to ischemic conditions. Synaptic terminals are the primary and early target in the development of damage to postischemic neural cells [26]. Disruption of ion transport and depolarization of presynaptic membranes leads to excessive release of glutamate from synaptic terminals into the intercellular space, which leads to metabolic disruption and triggers cell death in neurons of the CA1 field [15,27].

Despite the importance of damage to the hippocampus, such simulations of damage in cardiac arrest in humans and the timing of these lesions are poorly documented. Most studies report neuronal loss in the CA1 field of the hippocampus, enhancement of neurogenesis, astrogliosis and inflammation [16,17,28,29,30,31]. Demyelination in the three-vessel model of GCI has not yet been described. The study aimed to assess demyelination in the hippocampal layers and closely related neuronal loss, neurodegeneration, neuroinflammation, astrogliosis and oligodendrogenesis in the hippocampus on the three-vessel model of GCI. Although the study focused more on the assessment of damage to the hippocampus, signs of neurodegeneration were assessed in the neocortex and thalamus adjacent to the hippocampus.

## 2. Results

### 2.1. Survival and Neurological Deficit

The survival and neurological scores of animals after ischemia are presented in Table 1 and corresponded to those shown earlier for this model [16,19]. Mostly, animals died during the first hour after GCI, and the surviving animals showed a severe neurological deficit with manifestations such as the absence of reflexes, spastic paralysis and tonic tension of the muscles of the trunk. The severe neurological deficit after GCI persisted up to the endpoint, slightly decreasing from Day 10 to Day 30 (statistically insignificant). At Day 30 neurological scores of positive controls slightly decreased, but not significantly compared with an earlier time point. Sham operated animals showed 100% survival after surgery and no dysfunctions of the central nervous system.

### 2.2. Neuronal Loss and Neurodegeneration in the Hippocampus, Neocortex and Thalamus

**Figure 1:**
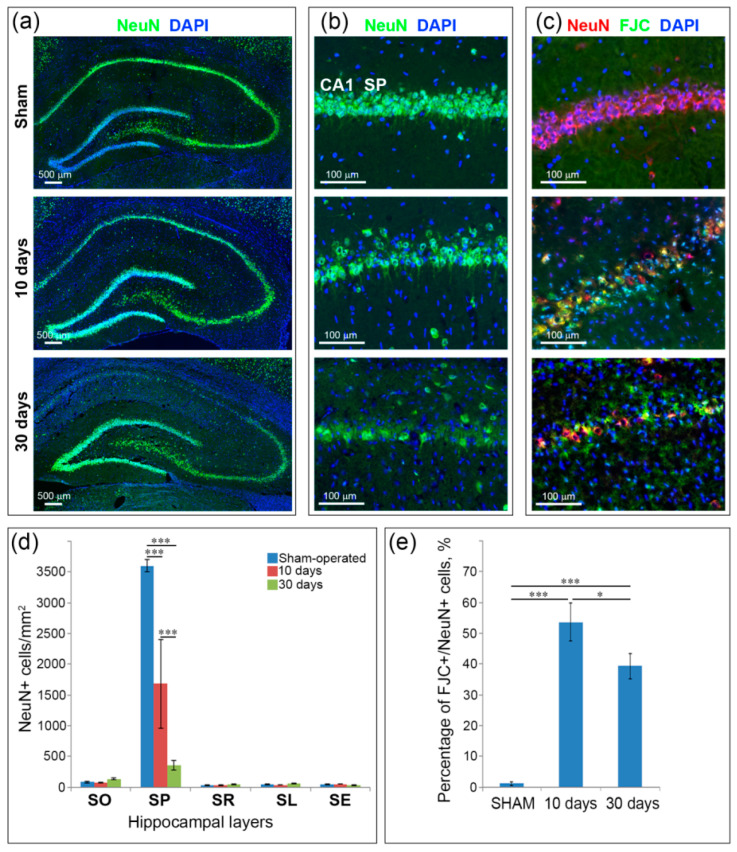
Neuronal loss in the hippocampus 10 and 30 days after global cerebral ischemia (GCI). (**a**) Example microphotographs of neuronal (NeuN)-positive neurons in the hippocampus of sham-operated controls and animals 10 and 30 days after GCI; (**b**,**c**) magnified views of NeuN-positive (**b**) and double-labeled Fluoro-Jade C (FJC)\NeuN-positive (**c**) neurons in the CA1 field of the hippocampus of sham-operated controls and animals 10 and 30 days after GCI; (**d**) comparison of NeuN+ cells in the CA1 field between sham-operated controls and animals 10 and 30 days after GCI; (**e**) percentage of FJC+\NeuN+ cells from NeuN-positive neurons in the CA1 field in sham-operated controls and animals 10 and 30 days after GCI. Significant differences relative to the sham-operated group, according to ANOVA after LSD correction for multiple comparisons: *—*p* < 0.05, ***—*p* < 0.001.

The most prominent neuronal loss was observed in the CA1 layer of pyramidal neurons. On Day 10 after GCI, the number of NeuN+ cells in the CA1 field decreased significantly (*p* < 0.001) compared to the sham-operated animals only in SP (Figure 1c). The layer appears rarefied with the integration of NeuN-negative small-nuclear cells, probably, activated microglia as revealed by previous studies [17,32]. On Day 30 after GCI in the CA1 field SP the number of NeuN+ cells significantly decreased both in comparison with the sham-operated control and the 10-day group (Figure 1b). At this time point only separate pyramidal NeuN+ neurons are observed. Some pyramidal neurons are very weakly stained (Figure 1a). On average, the number of pyramid neurons in SP 30 days after 7 min of GCI decreased by about 14 times (from 3538 cells per mm^2^ in the sham-operated animals to 245 cells per mm^2^ in animals from the 30-day group). Other layers of the hippocampus in the CA1 field did not show significant difference compared with controls in the number of NeuN+ cells (Figure 1b).

A combination of Fluoro-Jade C (FJC) and NeuN staining showed that 53.4% remaining pyramidal neurons in the hippocampus on Day 10 after GCI undergo degeneration (Figure 1c,e). On Day 30 the percentage of double-labeled FJC+\NeuN+ cells from surviving neurons was significantly (*p* < 0.05) reduced compared to the 10-day group, but still remain high (39.2%). In addition, neurodegeneration was observed in the neocortex (Figure S1) and thalamus (Figure S2). On Day 10 after GCI in the motor and somatosensory cortex small amount of degenerating neurons was found only in layers II–III. On the 30th day after GCI neurodegeneration was observed not only in layers II–III, but also extended to layer IV. In the thalamus, few degenerating neurons only in the paraventricular thalamic nucleus were found on Day 10 after ischemia. At the 30-Day time point neurodegeneration involved, in addition to the paraventricular thalamic nucleus, neighboring mediodorsal, intermediodorsal thalamic nuclei and habenular nucleus.

### 2.3. Inflammation and Specific Changes of Microglial Morphology after GCI

**Figure 2:**
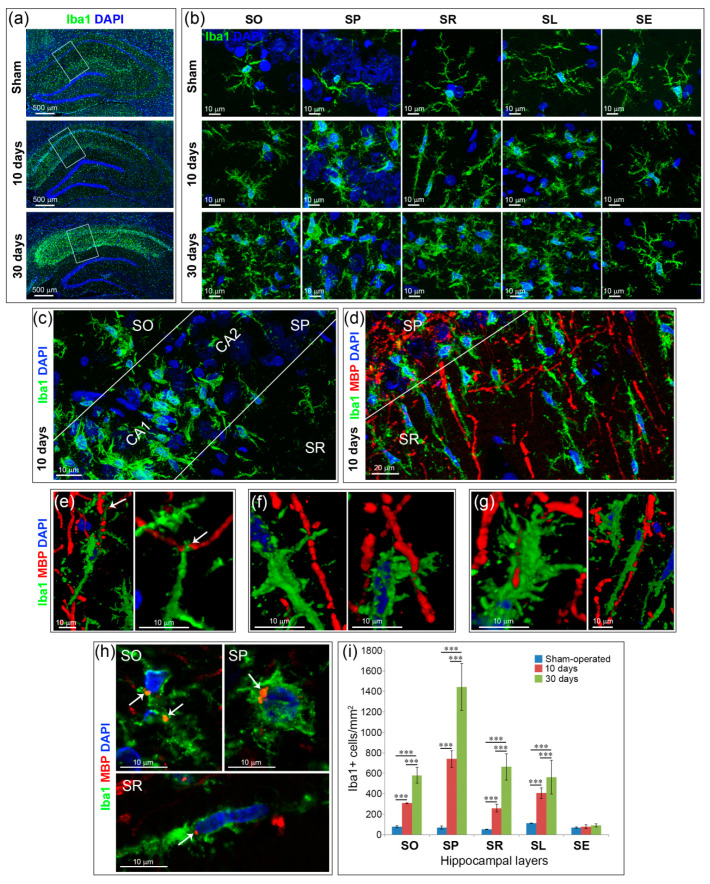
Microglia/macrophages (Iba1 + calls) in the hippocampal layers at the CA1 field level in rats after GCI (10 and 30 days) and the sham-operated animals. (**a**) General views of the hippocampus. The white line marks the area within the hippocampus where the cells with characteristic morphology of each layer were selected, 20× objective; (**b**) representative views of the morphology of Iba1+ cells in the hippocampal layers. Images taken from a series of optical slices using maximum intensity projection (MIP) of the z-stack; (**c**) CA1 and CA2 fields within the stratum pyramidale (SP) layer sharply differ in the number and immunoreactivity of Iba+ cells. The MIP image; (**d**) Fragment of field CA1 with rod-shaped microglia in stratum radiatum (SR); (**e**–**g**) types of interaction of Iba1+ cells with myelinated fibers in the SR, 10 days after GCI. 3D reconstruction of z-stacks. White arrows in (**e**) (left—general plan, right—enlarged fragment) indicate the place of contact of Iba1 + cells with myelinated axon; (**h**) myelin basic protein (MBP)-positive material (white arrows) is utilized by Iba1 + cells in the stratum oriens (SO), stratum pyramidale (SP) and substratum radiatum (SR); (**b**–**h**) 40× oil immersion objective; (**i**) quantification of Iba1+ cells in the hippocampal layers in the sham-operated and postischemic animals, at Day 10 and 30 after GCI. Significant differences between the groups, according to ANOVA after LSD correction for multiple comparisons: ***—*p* < 0.001.

In animals that underwent GCI, an accumulation of activated microglia was observed in all hippocampal layers at the CA1 field level except the SE (Figure 2a). On the 10th day after GCI in SO, the processes of the cells became thicker in a large part of the cells; the number of the processes increased. In the SP and SL, all Iba1+ cell bodies became larger, the processes were shorter and thicker. In the SP, the accumulation of Iba+ cells is limited to the CA1 field, where they are located everywhere between the pyramidal neurons (Figure 2b,c). In the SR, cells acquired a distinct rod-like morphology: a strongly elongated cell body and an elongated nucleus; numerous short processes depart from the cell body (Figure 2b). Double immunostaining with the myelin basic protein (MBP) and Iba1 showed that the elongated microglial bodies in the SR are oriented predominantly in the same direction as the myelinated fibers in this layer (Figure 2d).

**Figure 3:**
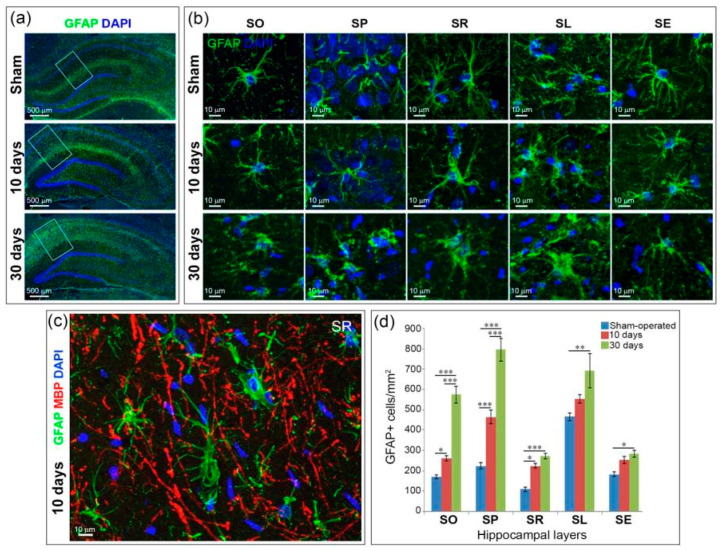
Astrogliosis and astrocyte morphology (GFAP+cells) after GCI. (**a**) General views of the hippocampus in sham-operated animals and 10 and 30 days after GCI. The white rectangles show the areas within which cells with morphology characteristic of each layer were selected, 20× objective; (**b**) representative views of the morphology of GFAP-positive cells in hippocampal layers. Images taken from a series of optical slices using software MIP; (**c**) An example of radial orientation of astrocyte processes in the SR. A 40× oil immersion objective was used to obtain (**b**) and (**c**) images; (**d**) quantification of GFAP-positive astrocytes in the hippocampal layers in the sham-operated and postischemic animals, at day 10 and 30 after GCI. Significant differences between the groups, according to ANOVA after LSD correction for multiple comparisons: ***—*p* < 0.001; **—*p* < 0.01; *—*p* < 0.05.

We identified three types of contacts of Iba1+ cells with myelinated axons in the SR: (1) elongated rod-shaped bodies of microglia binds fibers to the bipolar ends of their bodies (Figure 2e); (2) the lateral processes of microglia contacts with the myelinated fiber, crossing it at the nodes of Ranvier (Figure 2f); (3) Iba1+ cells enfold myelin fibers and extend their bodies along them (Figure 2g). Fragments of the MBP+ material can be found in the bodies of Iba1+ cells in the SO, SP and SR (Figure 2h).

On the 30th day after GCI in all layers of the hippocampus, except the SE, Iba1+ cells showed activated morphology with hypertrophied cell bodies and short thick processes. Quantification of the number of Iba1+ cells in the hippocampal layers showed that on the 10th and 30th day after GCI, the number of cells in all layers (SO, SP, SR, SL) increased significantly (*p* < 0.001) compared to the control, except the SE (Figure 2i). Thus, after both 10 and 30 days after GCI, only the SE Iba1+ cells did not respond to ischemic damage: their number and morphology did not change compared to the data in the control group of animals (Figure 2b,i).

### 2.4. Astrogliosis

The GCI-induced astrogliosis in the hippocampus is observed at Day 10 and continues to the 30th day in postischemic animals (Figure 3a). A significant part of GFAP-positive cells in hippocampal layers of postischemic animals showed morphologic changes characteristic of activated astrocytes, such as an enlarged body, thickening of the processes and an increase in their number (Figure 3b). Astrocyte activation after GCI, however, was not observed in all hippocampal layers. Ten days after GCI, no significant changes in GFAP-positive cell morphology were observed in the hippocampus layers of SO, SP, SE, but activated astrocytes with hypertrophied bodies and thickened processes appeared in SR and SL (Figure 3b). The astrocytes located between the myelin fibers in the SR maintained the radial orientation of their processes (Figure 3c). At the same time point, the number of astrocytes significantly increased when compared to the control group in the layers of SO, SP and SR (*p* < 0.05–0.001). The number of astrocytes in SL and SE tended to increase, but not statistically significantly (Figure 3d). At Day 30 after GCI, astrocytes cells in all layers of the hippocampus except SE had hypertrophic bodies and short processes, especially in the SR and SL layers (Figure 3b). Their number significantly increased compared with the sham-operated group in all hippocampal layers (*p* < 0.05–0.001) (Figure 3d). In sham-operated rats, astrocyte morphology remained unaffected: a small cell body with long thin processes.

### 2.5. GCI Causes Myelin and Oligodendrocyte Loss in the SO, SP and SR the Hippocampus

**Figure 4:**
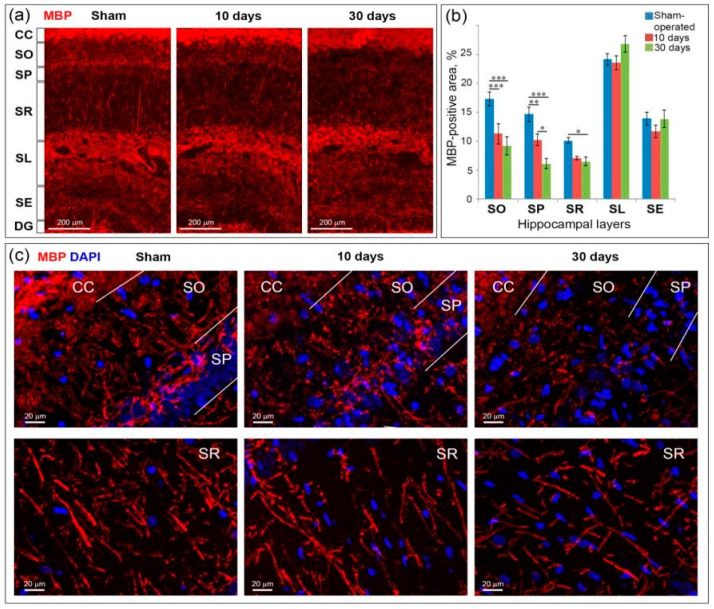
Myelin loss in the hippocampus at Days 10 and 30 after GCI. (**a**) General views of MBP-stained hippocampus at the CA1 field level at Days 10 and 30 after GCI and in sham-operated controls. CC—corpus callosum, SO, SP, SR, SL, SE—hippocampal layers, DG—dentate gyrus, 20× objective; (**b**) comparison of myelin content between the groups according to the percentage of MBP staining area. Significant differences between the groups, according to ANOVA after LSD correction for multiple comparisons: ***—*p* < 0.001; **—*p* < 0.01; *—*p* < 0.05; (**c**) MIP images of magnified fragments of the hippocampal layers, in which significant changes in the number of MBP after GCI were revealed. 40× oil immersion objective.

GCI caused myelin loss in all hippocampal layers excluding the SL end SE. The most prominent changes in myelination (MBP-positive area) after GCI were found in the SO and SP (Figure 4a,c). In the sections obtained both at Days 10 and 30 after GCI observed a decrease in the number of myelinated fibers, especially at the top part of the SP. The SR was also affected by GCI: in postischemic animals, myelinated fibers in this layer became fragmented. In sham-operated animals, most of the myelinated fibers in the SR were parallel to each other and perpendicular to the SP. Separate fibers can be traced throughout the entire SR, from the SP to SL, unlike the myelin fibers obtained from postischemic animals. A quantitative comparison of animal groups confirms that the SO and SP are most affected by GCI among hippocampal layers. In the SO and SP, the significant differences in the MBP-positive area were found both between the control and the 10-day time point and between the control and the 30-day time point. In addition, a significant (*p* < 0.05) decrease in the MBP-positive area on the 30th day after ischemia was found in the SR layer compared to the control (Figure 4b). Changes in the SL and SE layers were not statistically significant. Thus, in conditions of complete interruption of the cerebral blood flow followed by reperfusion, demyelination occurs in the hippocampal layers adjacent directly to the CA1 field that contains the processes of pyramidal neurons, the most vulnerable to ischemia.

**Figure 5:**
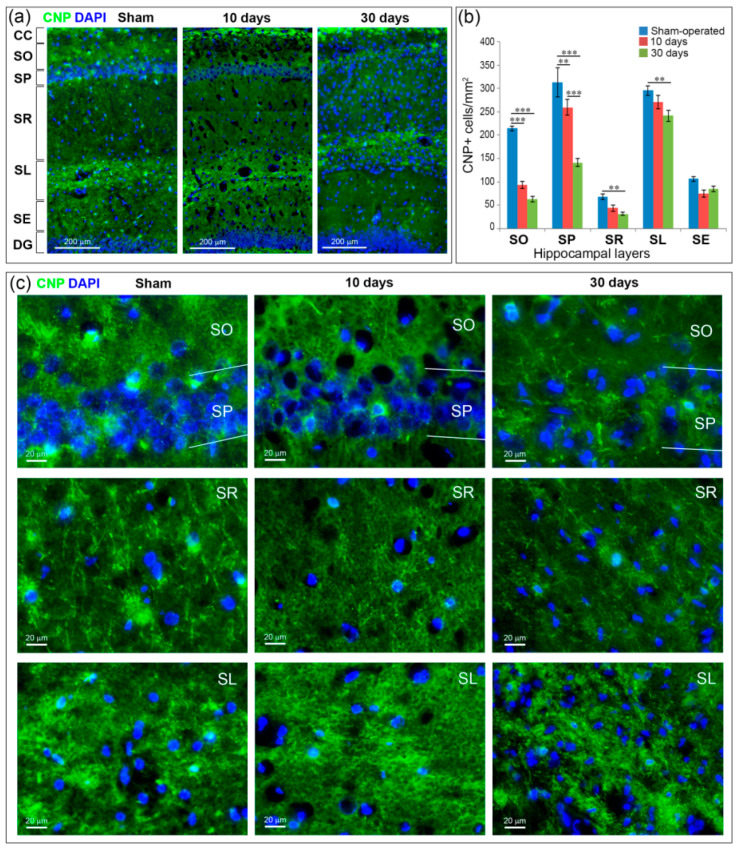
Myelinating oligodendrocytes (CNP+ cells) in the hippocampus at Days 10 and 30 after GCI. (**a**) General views of CNP-stained hippocampus at the CA1 field level at Days 10 and 30 after GCI and in sham-operated controls. CC—corpus callosum, SO, SP, SR, substratum lacunosum (SL) and substratum eumoleculare (SE)—hippocampal layers, DG—dentate gyrus, 20× objective; (**b**) comparison of CNP-positive cells between the groups. Significant differences between the groups, according to ANOVA after LSD correction for multiple comparisons: ***—*p* < 0.001; **—*p* < 0.01; (**c**) Magnified fragments of the hippocampal layers, in which significant changes in the number of MBP after GCI were revealed. 20× objective.

The number of myelinating oligodendrocytes also in the hippocampus was reduced after GCI (Figure 5). On Day 10 after GCI, similar to the change in myelin content, a significant decrease in the number of myelinating oligodendrocytes compared to the control was observed in the SO and SP hippocampal layers (*p* < 0.01). On Day 30 after GCI, the number of oligodendrocytes in the SO and SP layers was reduced both in comparison to the control (*p* < 0.001) and in comparison to the 10-day point (only the SP layer, *p* < 0.01). In addition, on Day 30, a significant reduction in the number of oligodendrocytes was observed in the SR and SL layers.

### 2.6. GCI Increases the Number of Immature Oligodendrocytes in the Hippocampus

**Figure 6:**
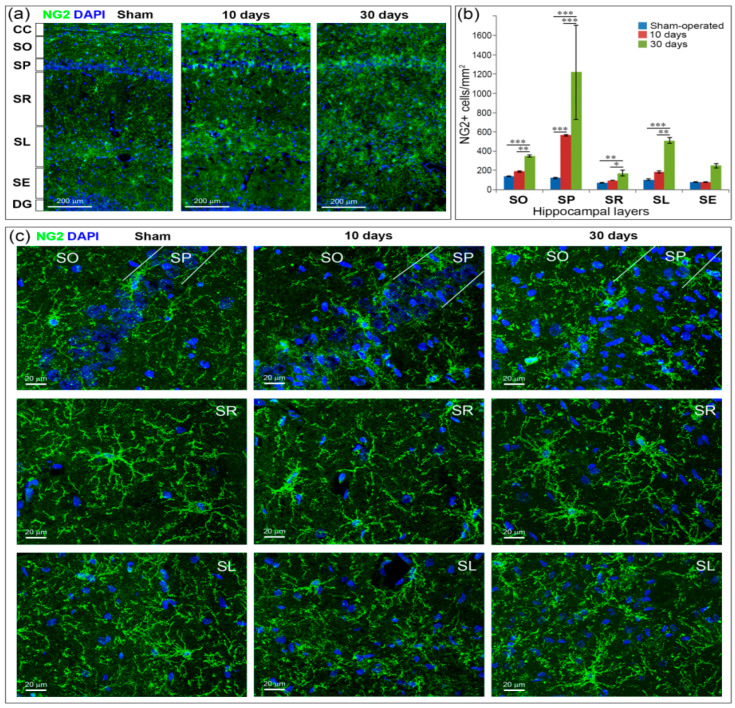
Oligodendrocyte precursors (OPCs) (NG2-positive cells) in the hippocampus 10 and 30 days after GCI. (**a**) Example microphotographs of the hippocampus at the CA1 field level in sham-operated controls and animals 10 and 30 days after GCI. CC—corpus callosum, SO, SP, SR, SL, SE—hippocampal layers, DG—dentate gyrus, 20× objective; (**b**) comparison of NG2-positive cells content between the groups. Significant differences between the groups, according to ANOVA after LSD correction for multiple comparisons: ***—*p* < 0.001; **—*p* < 0.01; *—*p* < 0.05; (**c**) MIP images of magnified fragments of the SO, SP, SR and SL hippocampal layers at the CA1 field. 40× oil immersion objective.

In an intact brain, OPCs (NG2-positive cells) in the hippocampus have many branched, radially sprouting processes. The morphology of NG2+ cells after GCI did not change as noticeably as microglia and astrocytes activated by GCI (Figure 6c). 10 days after GCI, NG2+ cells were not as multiprocessed as in the control group and had larger cell bodies. On Day 30 after GCI on the contrary, they were distinguished by a large number of processes and, in general, looked larger, especially in the SR and SL. Quantification of the NG2+ cells showed a significantly increased number of OPCs after GCI in all hippocampal layers except the SE (Figure 6a,b). 10 days after GCI, the number of immature oligodendrocytes increased compared to control only in the SP (*p* < 0.001). 30 days after GCI, the number of NG2+ cells significantly exceeded both the control and the 10-day time point in the SO (*p* < 0.001 and *p* < 0.01, respectively), SP (*p* < 0.001), SR (*p* < 0.01 and *p* < 0.05, respectively) and SL (*p* < 0.001 and *p* < 0.01, respectively) (Figure 6b).

## 3. Discussion

Our results for the first time have demonstrated myelin and oligodendrocyte loss of the hippocampus on the rat model of GCI, which implies complete interruption of cerebral blood flow and simulates cardiac arrest in humans. We investigated the dynamics of neuronal loss, neurodegeneration, astrogliosis, inflammation, utilization of disrupted myelin by microglia/macrophages and specific rod-shaped changes of microglial morphology.

To the best of our knowledge, demyelination has not been studied in a rat model of GCI to date. Earlier Lee et al. [33] in the gerbil model of GCI found myelin loss in the hippocampus, which started from 4 days after ischemia. Our results show that myelin and oligodendrocyte loss was observed 10 days after GCI and intensified by the 30th day in the hippocampal layers adjacent to the pyramidal neurons in the CA1 field (SO and SR), as well as the SP layer itself. Unlike focal ischemia, when there is an interruption of the blood supply to a limited area, global cerebral ischemia leads to a temporary cessation of blood supply to the entire brain. However, short-term cessation of blood flow leads to serious damage to only certain brain regions. A distinctive feature of the global ischemia-hypoxia model in rodents is severe damage to the hippocampal pyramidal neurons in the CA1 field [13,14,31,34], which is confirmed by the results of our previous [17,19,35] and present studies. In addition, a number of studies have found damage to the neurons of the cerebellum, cortex, striatum and thalamus in global ischemia-hypoxia [36,37,38,39]. Our study also found neurodegeneration in the neocortex and thalamus, slight on the 10th day after ischemia and increasing by the 30th day. The hippocampal subfields respond differently to GCI: pyramidal neurons in the CA3 field are relatively resistant to transient global cerebral ischemia, while neurons in the CA1 field are more susceptible to hypoxia. The death of neurons in this area is called the “delayed hippocampal neuronal death” [13,14], noticeable 4–7 days after GCI in gerbils [33,40]. Lee et al. [33] found an almost complete loss of hippocampal pyramidal neurons in the CA1 field in gerbils as early as 4 days after GCI. Our results demonstrated more delayed neuronal death in the rat model of GCI: we found the death of approximately half of neurons on the 10th day and more than 90% of neurons on the 30th day after GCI the CA1 field, whereas the CA2 and CA3 fields were less affected. The higher sensitivity to ischemia of the CA1 pyramidal neurons in gerbils compared to rats may be associated with increased NO synthesis [41] or metabolic features of GABAergic pyramidal neurons in the hippocampus, which is associated with the propensity of this species to epilepsy [42,43].

Apparently, pyramidal neurons of the CA1 field die mainly by the mechanism of glutamate-dependent excitotoxicity [44], since they receive a huge number of exciting glutamate inputs, which is dozens of times more than the number of inhibitory GABA inputs [45]. Main inputs are represented by afferents from the entorhinal cortex as part of alvear and performant pathways [46], Schaffer collaterals and numerous inputs from GABAergic interneurons within the hippocampus, among which up to 12 subtypes are distinguished [47]. The hippocampal network is very complex and is still the subject of morphologic studies [47,48]. One pyramidal neuron of the CA1 field can receive more than 30 thousand inputs in different layers of the hippocampus, including the SO, SP, SR and SL [45,48]. Among these numerous inputs the long axons from entorhinal cortex within alvear and perforant are myelinated, located in all layers of the hippocampus except the SE and having contacts with the basal and apical dendrites of the CA1 pyramidal neurons [49]. In addition, the SO and SP contain the myelinated axons of the CA1 pyramidal neurons that send fibers to the subiculum, entorhinal cortex and other extrinsic brain structures [50]. The Schaffer collaterals within the SL and their branches in the SR, which contacted with apical dendrites of the CA1 pyramidal neurons, are largely unmyelinated or weakly myelinated [51].

According to our results, on Day 10 after GCI significant myelin and oligodendrocyte loss was found only in the SO and SP. By the 30th day, myelin loss in these layers intensified and affected the SR, the number of oligodendrocytes decreased in the SR and SL. It is likely that earlier changes (Day 10) were found to be associated with axonal myelin destruction of dead pyramidal neurons of the CA1 field, while later changes (30 days) also affect the demyelination of afferents from alveus and the perforant pathway in the SO, SP and SR layers.

These assumptions are confirmed by phagocytosis of myelin and the time course of the detected morphologic changes in glial cells in the above layers of the hippocampus. In addition to the expected activation and increase in the number of astrocytes and microglia in these layers, which is consistent with numerous studies [52,53,54], we observed specific morphologic changes in these cells in the SR on the 10th day after GCI, associated with the orientation and extension of their bodies parallel to myelinated fibers and perpendicular to the layer of pyramidal neurons.

A morphology similar to that which we observed in the SR 10 days after ischemia–an elongated rod-shaped nucleus and a body with short processes, is characteristic of rod-shaped microglia [55,56,57,58]. Rod-shaped microglia have been described in the cortex in rats with diffuse brain injury [56], in the hippocampus in older adults [58] and present in experimental models of optic nerve degeneration and in some slowly developing neurodegenerative diseases progressing to dementia [55]. Individual cells or chains from rod microglia are adjacent to neuronal processes. It was shown that postischemic changes in the SR layer of the hippocampus are limited to the region of synaptic terminals (clumping or dispersion of the synaptic vesicle pools and damage to synaptic membranes) and synaptic terminals are the primary and early target in the development of damage to the postischemic neurons [14,26]. It is suggested that a close relationship with the processes of neurons reflects the participation of Rod microglia in synaptic stripping and neuronal circuitry reorganization [56,59]. The study of microglia-synaptic interactions showed that there is not complete destruction of the synapses, but selective partial phagocytosis or trogocytosis, of presynaptic structures [60]. The cell bodies of rod microglia, which we observed in the SR 10 days after GCI, spread along myelinated axons and made contact with them by their short processes. We suggest that together with the utilization of myelin and other cellular components, the rod microglia in this layer “disconnects” the excitatory inputs of pyramidal neurons from the afferents of the alvear and performant pathways to prevent neuronal loss due to glutamate excitotoxicity. With continued neuroinflammation on the 30th day after GCI, an accumulation of activated microglia, mainly with large round cell bodies and short processes, was observed in the SR and the phagocytosis occurs more actively. This corresponds to a further decrease in the MBP- positive area in the SR at Day 30 after GCI.

An interesting observation was the appearance of different types of contacts of rod-shaped microglia with myelinated axons including contacts by the bipolar ends of microglial bodies, by the microglial lateral processes and close grasping contact of microglial bodies with myelin fibers. Recent studies reveal that microglia and astrocytes play a major role in network formation during early development, in adulthood and neurodegeneration by directly pruning redundant synapses [61]. Microglia are also able to act as a sensor of synaptic activity and play a key role in the homeostatic regulation of neural excitation [62,63,64]. We assume that the bipolar contacts of rod-microglia bodies with myelinated fibers that we found, as well as the contacts of the lateral processes in the nodes of Ranvier, act as sensors for searching for synapses with excessive excitation and for pruning of such a contact. Similar pruning of superfluous excitatory synapses is shown during neurodevelopment [65]. Verification of these assumptions, as well as the functional significance of the third type of contacts, which is a close grasping contact of microglial bodies with myelin fibers, is the subject of further research.

According to our results, in parallel with ongoing myelin and oligodendrocyte loss on the 30th day after ischemia, remyelination processes begin, as evidenced by an increase in the number of OPCs in the hippocampus. A significant increase in the number of OPCs in the SO, SP and SR layers of the hippocampus, the most subjected to demyelination after GCI, indicates the beginning of the regenerative process of remyelination. The number of OPCs in these layers significantly increases by the 10th day and multiplicatively increases on the 30th day after GCI. NG2+ glia is recognized as a separate glial cell population giving rise to oligodendrocytes [66]. In response to damage, NG2+ glial cells are not only able to proliferate and migrate to lesions, but also differentiate into oligodendrocytes, forming new myelin sheaths that wrap around damaged axons and lead to their functional recovery [67,68]. In addition, the results of genomic and epigenetic studies have shown that reactive NG2 glia can also differentiate into GFAP-labeled astrocytes and DCX-expressing immature neurons after traumatic injury [69,70,71].

## 4. Materials and Methods

### 4.1. Animals and Housing

The study was performed on adult male Wistar rats weighing 250–300 g (*n* = 29; 15 rats survived after surgery) obtained from the vivarium of the E.D. Goldberg Institute of Pharmacology and Regenerative Medicine, Tomsk, Russia. Experiments were carried out in accordance with the rules adopted by the European Convention for the Protection of Vertebrate Animals used for Experimental and other Scientific Purposes. The study was approved on 22 March 2012 by the Animal Care and Use Committee at the E.D. Goldberg Institute of Pharmacology and Regenerative Medicine (protocol #22032012). Animals were housed in groups of seven animals per cage with about 300 cm^2^ per animal under standard conditions (12/12-h light/dark cycle, temperature of 22 ± 2 °C, humidity of 60%). Standard rodent chow (PK-120-1, Laboratorsnab, Ltd., Moscow, Russia) and water were provided ad libitum.

### 4.2. Experimental Design

Animals were randomly divided into two groups: sham-operated animals (*n* = 5) and animals, which underwent GCI (*n* = 10). Acute global cerebral ischemia was induced according to the new three-vessel model [16].

The surgery was performed as described previously [16,72]. Briefly, rats were anesthetized with chloral hydrate in a dose of 450 mg/kg intraperitoneally (Sigma-Aldrich Chemical Co., St. Louis, MO, USA) and placed on a homeothermic blanket (Temperature Control Unit HB 101/2, Letica Scientific Instruments, Barcelona, Spain) in a supine position. The body temperature was maintained at 37 °C. Cerebral blood flow was completely interrupted for 7 min by ligation of the major arteries supplying the brain (*truncus brachiocephalicus*, *arteria subclavia sinistra*, *and arteria carotis communis sinistra*). Access to *arteria carotis communis sinistra* was implemented through the ventral surface of the neck, while *truncus brachiocephalicus* and *arteria subclavia sinistra* were reached through the first intercostal space, bypassing the pleural cavity to avoid pneumothorax. The animals were intubated through the oral cavity. The same surgery was performed in sham-operated rats, but without ligation of blood vessels.

At Days 11 and 31 after surgery, neurological deficit was evaluated with the Stroke-index McGraw scale [72,73].

Animals were euthanized at Days 11 and 31 after surgery by transcardial perfusion with 4% paraformaldehyde under ether anesthesia. The brains were removed, fixed overnight with 4% paraformaldehyde solution, cryoprotected in sucrose phosphate buffer (24 h in 10% and 24 h in 20% solutions, respectively) at 4 °C, frozen in liquid nitrogen and stored at −80 °C for further immunohistochemical studies.

### 4.3. Immunochemistry and Microscopy

Coronal brain sections with 10-µm thickness were prepared using an HM525 cryostat (Thermo Fisher Scientific, Walldorf, Germany). Brain locations for immunohistochemical analysis were defined from −2.64 mm to −3.60 mm from bregma according to a rat brain atlas [74].

The following primary antibodies were used:

- rabbit-anti-NeuN (ABN78, Merck Millipore, Bedford, MA, USA) for detection of mature neurons;
- rabbit-anti-GFAP (ab7260, Abcam, Cambridge, MA, USA) for detection of astrocytes;
- rabbit-anti-Iba1 (019-19741, Wako Pure Chemical, Osaka, Japan) for detection of microglia/macrophages;
- rabbit-anti-NG2 (AB5320, Merck Millipore, Bedford, MA, USA) for detection of oligodendrocyte precursors (OPCs);
- mouse-anti-CNPase (MAB326, Merck Millipore, Bedford, MA, USA) for detection of myelinating oligodendrocytes;
- goat-anti-MBP (C-16, sc-13914, Santa Cruz Biotechnology, CA, USA) for myelin detection.

Additionally, double immunofluorescence was used for investigation of interaction of microglia and astrocytes with myelinated fibers. To assess neurodegeneration Fluoro-Jade C (FJC) staining was performed by combining with NeuN immunofluorescence according to manufacturer’s protocol (Fluoro-Jade^®^ C, Merck Millipore, Bedford, MA, USA). Primary antibodies were visualized by the following secondary antibodies: donkey-anti-goat AlexaFluor 594 (705–585–147, Jackson ImmunoResearch Laboratories, West Grove, PA, USA), donkey-anti-rabbit Alexa Flour 594 (711–585–152, Jackson ImmunoResearch Laboratories, West Grove, PA, USA) and donkey-anti-rabbit Alexa Fluor 488, (711–545–152, Jackson ImmunoResearch Laboratories, West Grove, PA, USA).

Brain sections were incubated with primary antibodies carried out overnight at 4 °C, with secondary antibodies–for 3 h at RT. Afterwards, stained brain sections were covered with antifade mounting medium Vectashield with DAPI (40,6-diamidino-2-phenylindole).

For each animal at least 4 microphotographs of the whole hippocampus of both (the left and right) hemispheres were obtained. Hippocampus general plans were photographed using an Axio Imager.Z2 (Carl Zeiss, Oberkochen, Germany) microscope (objective lens Plan-Apochromat 20×) and AxioVision 4.8 (Carl Zeiss) software with a MozaiX program module. 3D micrographs of separate groups of cells were obtained using Axio Imager Z1 (Carl Zeiss, Oberkochen, Germany) microscope (objective lens EC Plan-Neofluar 40× oil) with 3D ApoTome imaging system as a set of Z-stacks (45–50 sections per image), followed by the creation of a maximum intensity projection (MIP) image and 3D cell models.

### 4.4. Image Processing

**Figure 7:**
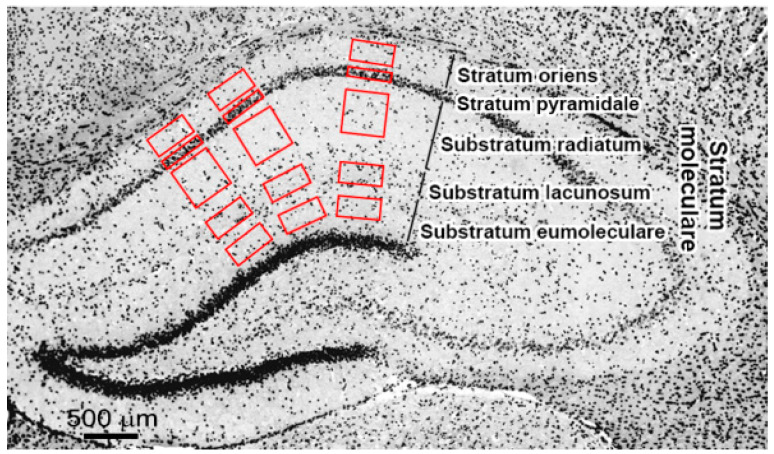
Scheme of calculation of NeuN+, GFAP+, Iba1+, NG2+ cells and MBP-positive area in layers of hippocampus counting to quantify severity of the ischemic lesion in experimental animals. The brain section painted DAPI (gray scale, invert color). Red boxes–regions of interest.

Calculation of NeuN+, GFAP+, Iba1+, NG2+, CNP+ cells was carried out using ImageJ software (National Institutes of Health, Bethesda, MD, USA). To quantify the severity of the ischemic lesion, cell count change in CA1 field of the hippocampus was evaluated by visual calculation of cells in the stratum oriens (SO), stratum pyramidale (SP), substratum radiatum (SR), substratum lacunosum (SL) and substratum eumoleculare (SE). Double-labeled FJC+\NeuN+ cells were calculated in the SP layer. The cells were counted within regions of interest (ROIs) according to the scheme presented in Figure 7. Three ROIs pre section, from 4 to 5 sections of the left and right hemispheres for each animal were analyzed (ROI: SO, SL, SE–500 × 250; SP–500 × 100; SR–500 × 500). Calculated number of cells per ROI were normalized to 1 mm^2^ area.

Myelin content in the hippocampal layers was assessed in ROIs similar to those for calculation of NeuN+, GFAP+, Iba1+, NG2+, CNP+ cells in the layers SO, SP, SR, SL and SE near the CA1 field (Figure 6). Assessment was performed using the Otsu thresholding method in the ImageJ (National Institutes of Health, Bethesda, MD, USA) implementation as a percent of MBP-positive area [35,75,76].

### 4.5. Statistical Analysis

Statistical analysis was performed using the Statistica 10.0 software (StatSoft, Inc., Tulsa, OK, USA). Mean values and standard errors of mean (SEM) for each type of labeled cells were calculated in the hippocampal layers around the CA1 fields: SO, SP, SR, SL and SE. Quantitative histology data were compared between the control, 10-day ischemic and 30-day ischemic groups using a two-way repeated measures analysis of variance (ANOVA) followed by post hoc least significant difference (LSD) tests for individual layers of the hippocampus. The statistical significance for all the analyses was less than 0.05.

## 5. Conclusions

Our study showed that GCI caused by a complete 7-min interruption of cerebral blood flow is accompanied not only by the loss of pyramidal neurons in the CA1 field, neurodegeneration of neocortex and thalamus, astrogliosis and inflammation in SP and adjacent layers of SO and SR, but also by myelin and oligodendrocyte loss in these layers of the hippocampus. At an earlier time-period postischemia (10 days), significant myelin and oligodendrocyte loss is observed in the SO and SP layers, and by 30 days it affects the SR layer, in which, presumably, an activated rod-like microglia first disconnects the contacts of the dying neurons and then utilizes myelin, axons, neurons and other cellular components. In parallel with the pathologic processes of neuronal death, demyelination and ongoing inflammation in the SO, SP and SR layers, the signs of restoration in the affected area of the hippocampus appear – a significant increase in the number of OPCs (NG2-positive cells) in the layers affected by GCI. Future long-term studies are needed to clarify the timeline of destruction and the degree of functional recovery of the hippocampal neural network after global brain ischemia that accompanies cardiac arrest. Another area of research may be related to elucidation of the functional role of rod-shaped microglia in the SR after GCI, which, probably, pruning excitatory synaptic contacts and remodel the hippocampal neural network to reduce neuronal loss.

## Figures and Tables

**Table 1:** Animal survival and neurological scores after surgery ^1^.

Group	Total Number	Survival, %		Neurological Scores	
		10 Days	30 Days	10 Days	30 Days
Sham-operated	10	100	100	0	0
Ischemia	19	52.6	47.4	6 (3–8) *	4 (2–9) *

^1^ significant differences compared with sham-operated animals: *—*p* < 0.01; neurological scores presented as median (range).

## Supplementary Materials

Supplementary materials can be found at https://www.mdpi.com/1422-0067/21/17/6246/s1. Figure S1: Neurodegeneration in the neocortex 10 and 30 days after GCI. Figure S2: Neurodegeneration in the thalamus 10 and 30 days after GCI.

## Abbreviations

GCI	Global cerebral ischemia
CA1, 2, 3	Subfields of the hippocampus
SO	Stratum oriens
SP	Stratum pyramidale
SR	Substratum radiatum
SL	Substratum lacunosum
SE	Substratum eumoleculare
NeuN	Neuronal nuclear antigen
FJC	Fluoro-Jade C
Iba1	Ionized calcium-binding adapter molecule 1
GFAP	Glial fibrillary acidic protein
MBP	Myelin basic protein
OPCs	Oligodendrocyte progenitor cells
CNP	2′,3′-Cyclic nucleotide 3′-phosphodiesterase
NG2	Neural/glial antigen 2
MIP	Maximum intensity projection
LSD	Least significant difference

## References

[1] Etherton M.R., Wu O., Giese A., Lauer A., Boulouis G., Mills B., Cloonan L., Donahue K.L., Copen W., Schaefer P. (2019). White Matter Integrity and Early Outcomes after Acute Ischemic Stroke. Transl. Stroke Res..

[2] Desmond D.W. (2002). Cognition and White Matter Lesions. Cerebrovasc. Dis..

[3] Simons M., Nave K.A. (2016). Oligodendrocytes: Myelination and axonal support. Cold Spring Harb. Perspect. Biol..

[4] Khodanovich M.Y., Kisel A.A., Akulov A.E., Atochin D.N., Kudabaeva M.S., Glazacheva V.Y., Svetlik M.V., Medvednikova Y.A., Mustafina L.R., Yarnykh V.L. (2018). Quantitative assessment of demyelination in ischemic stroke in vivo using macromolecular proton fraction mapping. J. Cereb. Blood Flow Metab..

[5] Dewar D., Underhill S.M., Goldberg M.P. (2003). Oligodendrocytes and ischemic brain injury. J. Cereb. Blood Flow Metab..

[6] Pantoni L., Garcia J.H., Gutierrez J.A. (1996). Cerebral white matter is highly vulnerable to ischemia. Stroke.

[7] Jing L., He Q., Zhang J.Z., Andy Li P. (2013). Temporal profile of astrocytes and changes of oligodendrocyte-based myelin following middle cerebral artery occlusion in diabetic and non-diabetic rats. Int. J. Biol. Sci..

[8] Han C.W., Lee K.H., Noh M.G., Kim J.M., Kim H.S., Kim H.S., Kim R.G., Cho J., Kim H.I., Lee M.C. (2017). An experimental infarct targeting the internal capsule: Histopathological and ultrastructural changes. J. Pathol. Transl. Med..

[9] McIver S.R., Muccigrosso M., Gonzales E.R., Lee J.M., Roberts M.S., Sands M.S., Goldberg M.P. (2010). Oligodendrocyte degeneration and recovery after focal cerebral ischemia. Neuroscience.

[10] Shi H., Hua X., Leak R., Shi Y., An C., Suenagac J., Chen J., Gao Y. (2016). Demyelination as a Rational Therapeutic Target for Ischemic or Traumatic Brain Injury. Physiol. Behav..

[11] Lee S., Lee M., Hong Y., Won J., Lee Y., Kang S.G., Chang K.T., Hong Y. (2014). Middle cerebral artery occlusion methods in rat versus mouse models of transient focal cerebral ischemic stroke. Neural Regen. Res..

[12] Khodanovich M.Y., Kisel A.A. (2015). Animal models of cerebral ischemia. AIP Conf. Proc..

[13] Kirino T., Tamura A., Sano K. (1984). Delayed neuronal death in the rat hippocampus following transient forebrain ischemia. Acta Neuropathol..

[14] Kirino T. (1982). Delayed neuronal death in the gerbil hippocampus following ischemia. Brain Res..

[15] Nitatori T., Sato N., Waguri S., Karasawa Y., Araki H., Shibanai K., Kominami E., Uchiyama Y. (1995). Delayed neuronal death in the CA1 pyramidal cell layer of the gerbil hippocampus following transient ischemia is apoptosis. J. Neurosci..

[16] Atochin D.N., Chernysheva G.A., Aliev O.I., Smolyakova V.I., Osipenko A.N., Logvinov S.V., Zhdankina A.A., Plotnikova T.M., Plotnikov M.B. (2017). An improved three-vessel occlusion model of global cerebral ischemia in rats. Brain Res. Bull..

[17] Khodanovich M., Kisel A., Kudabaeva M., Chernysheva G., Smolyakova V., Krutenkova E., Wasserlauf I., Plotnikov M., Yarnykh V. (2018). Effects of fluoxetine on hippocampal neurogenesis and neuroprotection in the model of global cerebral ischemia in rats. Int. J. Mol. Sci..

[18] Khodanovich M.Y., Kisel’ A.A., Chernysheva G.A., Smol’yakova V.I., Kudabaeva M.S., Krutenkova E.P., Tyumentseva Y., Plotnikov M.B. (2019). p-Tyrosol Enhances the Production of New Neurons in the Hippocampal CA1 Field after Transient Global Cerebral Ischemia in Rats. Bull. Exp. Biol. Med..

[19] Khodanovich M.Y., Kisel’ A.A., Chernysheva G.A., Smol’yakova V.I., Savchenko R.R., Plotnikov M.B. (2016). Effect of Fluoxetine on Neurogenesis in Hippocampal Dentate Gyrus after Global Transient Cerebral Ischemia in Rats. Bull. Exp. Biol. Med..

[20] Martínez N.S., Machado J.M., Saad H.P., Antich R.M.C., Acosta J.A.B., Salgueiro S.R., Illera G.G., Alba J.S., del Barco Herrera D.G. (2012). Global brain ischemia in Mongolian gerbils: Assessing the level of anastomosis in the cerebral circle of Willis. Acta Neurobiol. Exp. (Wars.).

[21] Squire L.R., Wixted J.T. (2011). The Cognitive Neuroscience of Human Memory Since H.M. Annu. Rev. Neurosci..

[22] Kim S., Dede A.J.O., Hopkins R.O., Squire L.R. (2015). Memory, scene construction, and the human hippocampus. Proc. Natl. Acad. Sci. USA.

[23] Kim S., Borst G., Thompson W.L., Hopkins R.O., Kosslyn S.M., Squire L.R. (2013). Sparing of spatial mental imagery in patients with hippocampal lesions. Learn. Mem..

[24] Zola-Morgan S., Squire L.R., Amaral D.G. (1986). Human amnesia and the medial temporal region: Enduring memory impairment following a bilateral lesion limited to field CA1 of the hippocampus. J. Neurosci..

[25] Rempel-Clower N.L., Zola S.M., Squire L.R., Amaral D.G. (1996). Three cases of enduring memory impairment after bilateral damage limited to the hippocampal formation. J. Neurosci..

[26] Ekstrom D.K.J., Diemer N.H. (1982). Complete cerebral ischaemia in the rat: An ultrastructural and stereological analysis of the distal stratum radiatum in the hippocampal CA-1 region. Neuropathol. Appl. Neurol..

[27] Benveniste H., Jorgensen M.B., Sandberg M., Christensen T., Hagberg H., Diemer N.H. (1989). Ischemic damage in hippocampal CA1 is dependent on glutamate release and intact innervation from CA3. J. Cereb. Blood Flow Metab..

[28] Tanaka R., Yamashiro K., Mochizuki H., Cho N., Onodera M., Mizuno Y., Urabe T. (2004). Neurogenesis after transient global ischemia in the adult hippocampus visualized by improved retroviral vector. Stroke.

[29] Nakatomi H., Nakatomi H., Kuriu T., Kuriu T., Okabe S., Okabe S., Yamamoto S.-I., Yamamoto S.-I., Hatano O., Hatano O. (2002). Regeneration of hippocampal pyramidal neurons afetr ischemic brain injury by recruitnt of endogenous neural progenitors. Cell.

[30] Kudabayeva M., Kisel A., Chernysheva G., Smol’Yakova V., Plotnikov M., Khodanovich M. (2017). The increase in the number of astrocytes in the total cerebral ischemia model in rats. J. Phys. Conf. Ser..

[31] Dohi K., Shioda S., Mizushima H., Homma H., Ozawa H., Nakai Y., Matsumoto K. (1998). Delayed neuronal cell death and microglial cell reactivity in the CA1 region of the rat hippocampus in the cardiac arrest model. Med. Mol. Morphol..

[32] Pforte C., Henrich-Noack P., Baldauf K., Reymann K.G. (2005). Increase in proliferation and gliogenesis but decrease of early neurogenesis in the rat forebrain shortly after transient global ischemia. Neuroscience.

[33] Lee J., Park J.H., Ahn J.H., Kim I.H., Cho J.H., Choi J.H., Yoo K., Lee C.H., Hwang I.K., Cho J.H. (2016). New GABAergic Neurogenesis in the Hippocampal CA1 Region of a Gerbil Model of Long-Term Survival after Transient Cerebral Ischemic Injury. Brain Pathol..

[34] Johansen F.F., Balslev Jørgensen M., Diemer N.H. (1983). Resistance of hippocampal CA-1 interneurons to 20 min of transient cerebral ischemia in the rat. Acta Neuropathol..

[35] Khodanovich M.Y., Pishchelko A.O., Glazacheva V.Y., Pan E.S., Krutenkova E.P., Trusov V.B., Yarnykh V.L. (2019). Plant polyprenols reduce demyelination and recover impaired oligodendrogenesis and neurogenesis in the cuprizone murine model of multiple sclerosis. Phyther. Res..

[36] Pulsinelli W.A., Brierley J.B. (1979). A New Model of Bilateral Hemispheric Ischemia in the Unanesthetized Rat. Stroke.

[37] Radovsky A.N.N., Katz L., Ebmeyer U.W.E., Safar P. (1997). Ischemic Neurons in Rat Brains After 6, 8, or 10 Minutes of Transient Hypoxic Ischemia. Toxicol. Pathol..

[38] Böttiger B.W., Schmitz B., Wiessner C., Vogel P., Hossmann K.A. (1998). Neuronal stress response and neuronal cell damage after cardiocirculatory arrest in rats. J. Cereb. Blood Flow Metab..

[39] Shoykhet M., Simons D.J., Alexander H., Hosler C., Kochanek P.M., Clark R.S.B. (2012). Thalamocortical dysfunction and thalamic injury after asphyxial cardiac arrest in developing rats. J. Neurosci..

[40] Wahul A.B., Joshi P.C., Kumar A., Chakravarty S. (2018). Transient global cerebral ischemia differentially affects cortex, striatum and hippocampus in Bilateral Common Carotid Arterial occlusion (BCCAo) mouse model. J. Chem. Neuroanat..

[41] Paschen W. (1995). Comparison of biochemical disturbances in hippocampal slices of gerbil and rat during and after in vitro ischemia. Neurosci. Lett..

[42] Scotti A.L., Kalt G., Bollag O., Nitsch C. (1997). Parvalbumin disappears from GABAergic CA1 neurons of the gerbil hippocampus with seizure onset while its presence persists in the perforant path. Brain Res..

[43] Kang T.C., Park S.K., Bahn J.H., Chang J.S., Cho S.W., Choi S.Y., Won M.H. (2001). Comparative studies on the GABA-transaminase immunoreactivity in rat and gerbil brains. Mol. Cells.

[44] Butler T.R., Self R.L., Smith K.J., Sharrett-field L.J., Berry J.N., Littleton J.M., Pauly J.R., Mulholland P.J., Prendergast A. (2010). Selective vulnerability of hippocampal cornu ammonis 1 pyramidal cells to excitotoxic insult is associated with the expression of polyamine-sensitive N-methyl- D-asparate-type glutamate receptors. Neuroscience.

[45] Megías M., Emri Z., Freund T.F., Gulyás A.I. (2001). Total number and distribution of inhibitory and excitatory synapses on hippocampal CA1 pyramidal cells. Neuroscience.

[46] Deller T., Adelmann G., Nitsch R., Frotscher M. (1996). The alvear pathway of the rat hippocampus. Cell Tissue Res..

[47] Migliore R., Lupascu C.A., Bologna L.L., Romani A., Courcol J.D., Antonel S., Van Geit W.A.H., Thomson A.M., Mercer A., Lange S. (2018). The physiological variability of channel density in hippocampal CA1 pyramidal cells and interneurons explored using a unified data-driven modeling workflow. PLoS Comput. Biol..

[48] Szilágyi T., Orbán-Kis K., Horváth E., Metz J., Pap Z., Pávai Z. (2011). Morphological identification of neuron types in the rat hippocampus. Rom. J. Morphol. Embryol..

[49] Meier S., Bräuer A.U., Heimrich B., Nitsch R., Savaskan N.E. (2004). Myelination in the hippocampus during development and following lesion. Cell. Mol. Life Sci..

[50] Arszovszki A., Borhegyi Z., Klausberger T. (2014). Three axonal projection routes of individual pyramidal cells in the ventral CA1 hippocampus. Front. Neuroanat..

[51] Andersen P., Silfvenius H., Sundberg S.H., Sveen O., Wigstrom H. (1978). Functional characteristics of unmyelinated fibres in the hippocampal cortex. Brain Res..

[52] Jørgensen M.B., Finsen B.R., Jensen M.B., Castellano B., Diemer N.H., Zimmer J. (1993). Microglial and Astroglial Reactions to Ischemic and Kainic Acid-Induced Lesions of the Adult Rat Hippocampus. Exp. Neurol..

[53] Sugawara T., Leẃn A., Noshita N., Gasche Y., Chan P.H. (2002). Effects of global ischemia duration on neuronal, astroglial, oligodendroglial, and microglial reactions in the vulnerable hippocampal CA1 subregion in rats. J. Neurotrauma.

[54] Sadelli K., Stamegna J.C., Girard S.D., Baril N., Escoffier G., Brus M., Véron A.D., Khrestchatisky M., Roman F.S. (2017). Global cerebral ischemia in rats leads to amnesia due to selective neuronal death followed by astroglial scar formation in the CA1 layer. Neurobiol. Learn. Mem..

[55] Holloway O.G., Canty A.J., King A.E., Ziebell J.M. (2019). Rod microglia and their role in neurological diseases. Semin. Cell Dev. Biol..

[56] Taylor S.E., Morganti-Kossmann C., Lifshitz J., Ziebell J.M. (2014). Rod microglia: A morphological definition. PLoS ONE.

[57] Au N.P.B., Ma C.H.E. (2017). Recent advances in the study of bipolar/rod-shaped microglia and their roles in neurodegeneration. Front. Aging Neurosci..

[58] Bachstetter A.D., Ighodaro E.T., Hassoun Y., Aldeiri D., Neltner J.H., Patel E., Abner E.L., Nelson P.T. (2017). Rod-shaped microglia morphology is associated with aging in 2 human autopsy series. Neurobiol. Aging.

[59] Zhu L., Wang L., Ju F., Ran Y., Wang C., Zhang S. (2017). Transient global cerebral ischemia induces rapid and sustained reorganization of synaptic structures. J. Cereb. Blood Flow Metab..

[60] Weinhard L., Di Bartolomei G., Bolasco G., Machado P., Schieber N.L., Neniskyte U., Exiga M., Vadisiute A., Raggioli A., Schertel A. (2018). Microglia remodel synapses by presynaptic trogocytosis and spine head filopodia induction. Nat. Commun..

[61] Henstridge C.M., Tzioras M., Paolicelli R.C. (2019). Glial contribution to excitatory and inhibitory synapse loss in neurodegeneration. Front. Cell. Neurosci..

[62] Ji K., Akgul G., Wollmuth L.P., Tsirka S.E. (2013). Microglia Actively Regulate the Number of Functional Synapses. PLoS ONE.

[63] Bechade C., Cantaut-belarif Y., Bessis A. (2013). Microglial control of neuronal activity. Front. Cell. Neurosci..

[64] Li Y., Du X., Liu C., Wen Z., Du J. (2012). Reciprocal Regulation between Resting Microglial Dynamics and Neuronal Activity In Vivo. Dev. Cell.

[65] Paolicelli R.C., Bolasco G., Pagani F., Maggi L., Scianni M., Panzanelli P., Giustetto M., Ferreira T.A., Guiducci E., Dumas L. (2011). Synaptic pruning by microglia is necessary for normal brain development. Science.

[66] Dawson M.R.L., Polito A., Levine J.M., Reynolds R. (2003). NG2-expressing glial progenitor cells: An abundant and widespread population of cycling cells in the adult rat CNS. Mol. Cell. Neurosci..

[67] Song F.E., Huang J.L., Lin S.H., Wang S., Ma G.F., Tong X.P. (2017). Roles of NG2-glia in ischemic stroke. CNS Neurosci. Ther..

[68] Zhao B., Zhao C.Z., Zhang X.Y., Huang X.Q., Shi W.Z., Fang S.H., Lu Y.B., Zhang W.P., Xia Q., Wei E.Q. (2012). The new P2Y-like receptor G protein-coupled receptor 17 mediates acute neuronal injury and late microgliosis after focal cerebral ischemia in rats. Neuroscience.

[69] Tatsumi K., Takebayashi H., Manabe T., Tanaka K.F., Makinodan M., Yamauchi T., Makinodan E., Matsuyoshi H., Okuda H., Ikenaka K. (2008). Genetic fate mapping of Olig2 progenitors in the injured adult cerebral cortex reveals preferential differentiation into astrocytes. J. Neurosci. Res..

[70] Komitova M., Serwanski D.R., Richard Lu Q., Nishiyama A. (2011). NG2 cells are not a major source of reactive astrocytes after neocortical stab wound injury. Glia.

[71] Heinrich C., Bergami M., Gascón S., Lepier A., Viganò F., Dimou L., Sutor B., Berninger B., Götz M. (2014). Sox2-mediated conversion of NG2 glia into induced neurons in the injured adult cerebral cortex. Stem Cell Reports.

[72] Chernysheva G.A., Smol’yakova V.I., Osipenko A.N., Plotnikov M.B. (2014). Evaluation of Survival and Neurological Deficit in Rats in the New Model of Global Transient Cerebral Ischemia. Bull. Exp. Biol. Med..

[73] McGraw C.P. (1977). Experimental Cerebral Infarction Effects of Pentobarbital in Mongolian Gerbils. Arch. Neurol..

[74] Paxinos G., Watson C. (2007). The Rat Brain in Stereotaxic Coordinates.

[75] Otsu N. (1979). A threshold selection method from gray-level histograms. IEEE Trans. Syst. Man Cybern..

[76] Ercan E., Han J.M., Di Nardo A., Winden K., Han M.-J., Hoyo L., Saffari A., Leask A., Geschwind D.H., Sahin M. (2017). Neuronal CTGF/CCN2 negatively regulates myelination in a mouse model of tuberous sclerosis complex. J. Exp. Med..

