# Supplementary material for: Neurodegeneration, Myelin Loss and Glial Response in the Three-Vessel Global Ischemia Model in Rat
Source: Int J Mol Sci. 2020 Aug 28;21(17):6246. doi: 10.3390/ijms21176246 (PMC7504277; doi:10.3390/ijms21176246)
Supplement: Supplementary file 1 [file ijms-21-06246-s001.pdf]

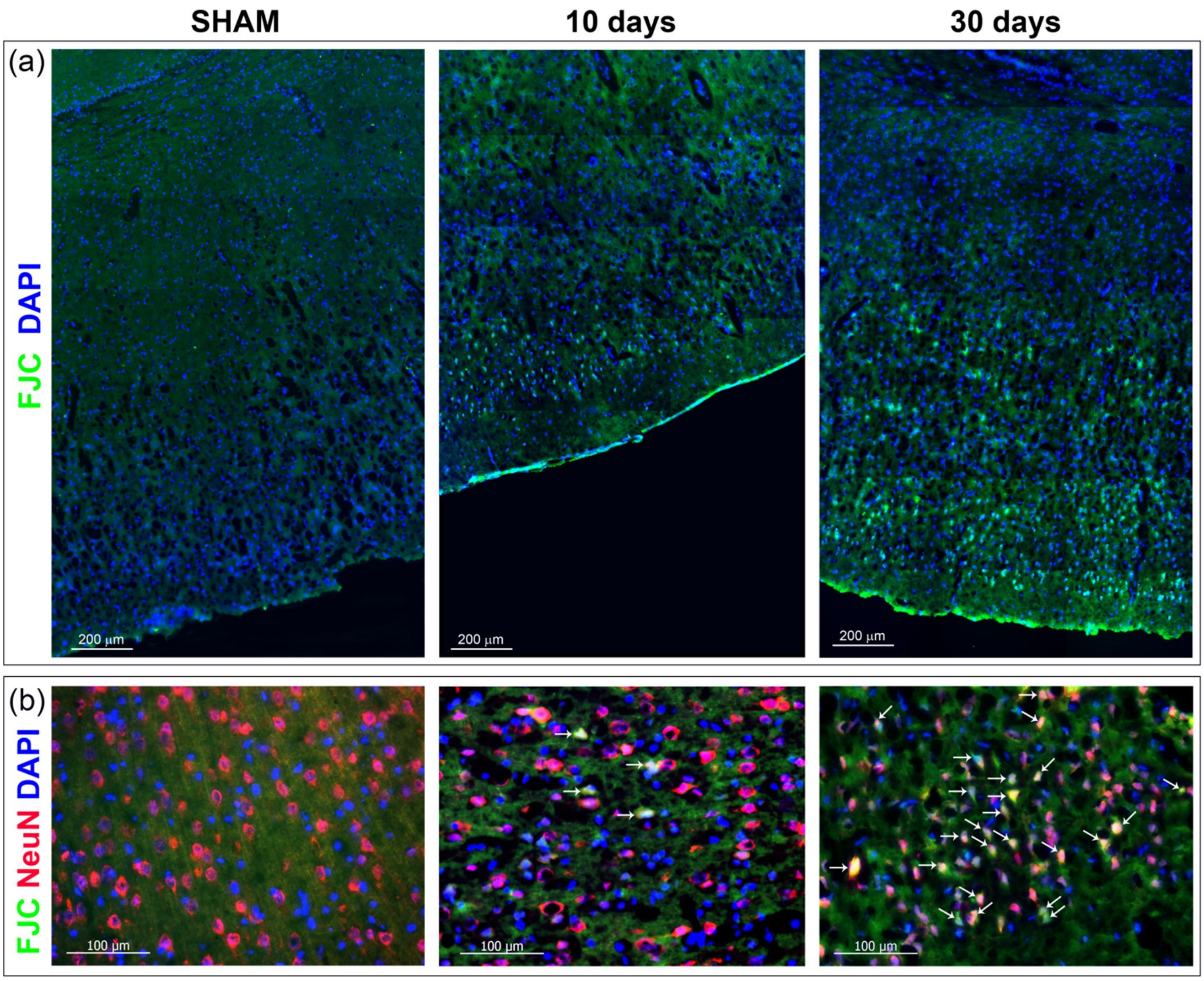

Figure S1. Neurodegeneration in the neocortex 10 and 30 days after GCI. (a) – Example microphotographs of FGC-positive degenerating neurons in the motor cortex of sham-operated controls and animals 10 and 30 days after GCI. (b) – Magnified views of double-labeled FGC+\NeuN+ cells in II cortical layer of sham-operated controls and animals in 10 and 30 days after GCI. The FGC+\NeuN+ cells are marked by arrows. 20× objective

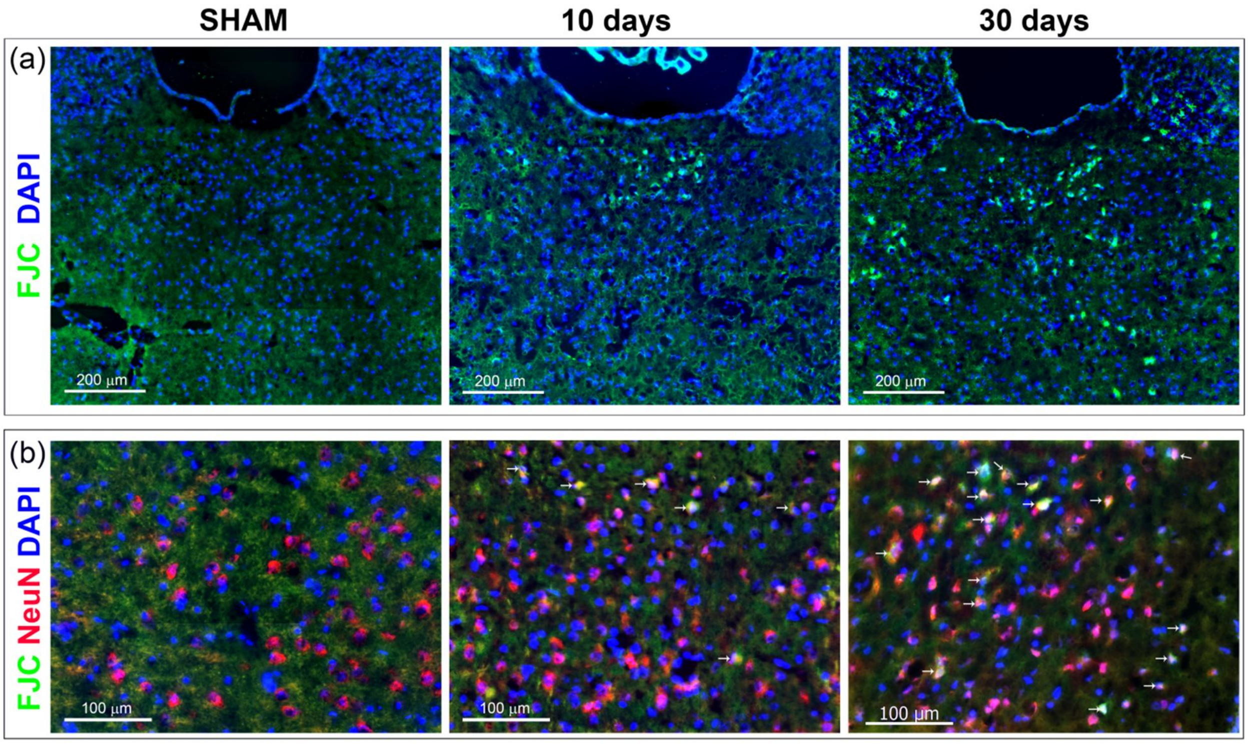

Figure S2. Neurodegeneration in the thalamus 10 and 30 days after GCI. (a) – Example microphotographs of FGC-positive degenerating neurons in the thalamus near the third ventricle of sham-operated controls and animals 10 and 30 days after GCI. (b) – Magnified views of double-labeled FGC+\NeuN+ cells in the paraventricular thalamic nucleus of sham-operated controls and animals in 10 and 30 days after GCI. The FGC+\NeuN+ cells are marked by arrows. 20× objective.
